# Eco-epidemiology of arbovirus infections among non-human primates in Southeastern Brazil

**DOI:** 10.1371/journal.pntd.0013743

**Published:** 2025-11-19

**Authors:** Leonardo La Serra, Rafael L. S. Cazarotti, Vitoria M. Scrich, Larissa M. Bueno, Andreia N. Carvalho, Daniel M. M. Jorge, Murilo H. A. Cassiano, Renan B. do Amaral, Soraya J. Badra, Gustavo R. Canale, Gilberto Sabino-Santos, Luiz T. M. Figueiredo

**Affiliations:** 1 Center for Virology Research, Ribeirão Preto Medical School, University of São Paulo, Ribeirão Preto, São Paulo, Brazil; 2 Environmental Sciences Graduate Program, Institute of Energy and Environment, University of Sao Paulo, Ubatuba, Brazil; 3 Department of Veterinary Medicine, University of São Paulo, Pirassununga, São Paulo, Brazil; 4 Department of Cellular and Molecular Biology and Pathogenic Bioagent, University of São Paulo, Ribeirão Preto, São Paulo, Brazil; 5 Department of Microbiology and Immunology, University of Michigan Medical School, Ann Arbor, Michigan, United States of America; 6 Institute of Natural, Human, and Social Sciences, Federal University of Mato Grosso, Sinop, Mato Grosso, Brazil; 7 Department of Microbiology & Immunology, Tulane University School of Medicine, New Orleans, Louisiana, United States of America; 8 Smithsonian Institution, National Zoo and Conservation Biology Institute, Front Royal, Virginia, United States of America; Solena Ag, UNITED STATES OF AMERICA

## Abstract

Orthoflaviviruses and alphaviruses are arboviruses responsible for human diseases in tropical and subtropical countries. We aimed to detect infections with arboviruses and to evaluate the ecological patterns related to these infections among non-human primates (NHPs) in southeastern Brazil. Of the 248 molecularly screened NHPs, 30 were infected with orthoflaviviruses, which highlighted hotspots of arboviruses. We identified genome fragments of orthoflaviviruses *Orthoflavivirus denguei* 1 (DENV-1), 2 (DENV-2) and 3 (DENV-3), *Orthoflavivirus louisense* (SLEV), *Orthoflavivirus zikaense* (ZIKV), and *Orthoflavivirus flavi* (YFV). No alphaviruses were detected. Amid a human outbreak of YFV, black-tufted marmoset (*Callithrix penicillata*) was identified as being infected. SLEV and ZIKV were found in saliva samples and rectal swabs obtained from NHPs, a potential route for non-vector transmission of these viruses. This is the first report of infection with SLEV in the golden-handed tamarin (*Saguinus midas*) as well as coinfections with ZIKV and DENV-3 in *C. penicillata* and with ZIKV and SLEV in black howler monkey (*Alouatta caraya*). The isolation of ZIKV and SLEV from the saliva of NHPs may suggest an alternative mechanism for the maintenance of these viruses within NHP communities, in addition to the conventional transmission by mosquitoes. These findings are fundamental to support public health policy decisions and to foster ongoing eco-epidemiological surveillance of arboviruses in the context of the human-animal interface.

## Author summary

Arboviruses pose a significant public health threat worldwide and underlie major infectious disease outbreaks. These viruses are transmitted by complex dynamics, which are influenced by various factors, including viral, ecological, and epidemiological factors, vectors, and human movement. However, an incomplete understanding of how natural hosts (especially mammals such as non-human primates (NHPs)) and certain orthoflaviviruses and alphaviruses are intricately related prevents outbreaks from being predicted and effective public health interventions from being implemented. In this study, we aimed to improve our understanding of host-vector dynamics by integrating knowledge from diverse disciplines, including virology, epidemiology, and ecological modeling. We were able to identify an undocumented orthoflavivirus in NHPs, *Orthoflavivirus louisense* (SLEV), and to correlate infections with geographical hotspots and specific NHP species. Employing a One Health eco-epidemiological approach was more suitable for surveilling orthoflavivirus outbreaks and could allow cost-effective public health interventions to be implemented through targeted mitigation strategies. Furthermore, we gained valuable insights into how orthoflaviviruses and less explored hosts are related, which is crucial knowledge for arbovirus surveillance and preservation of mammalian biodiversity worldwide, particularly in a climate change scenario.

## Introduction

Arboviruses (arthropod-borne viruses) account for many epidemics of human diseases and pose serious public health threats in tropical and subtropical regions of the world. In Brazil, arboviruses belonging to the family *Flaviviridae,* genus *Orthoflavivirus,* and the family *Togaviridae,* genus *Alphavirus*, have been of particular interest because they frequently cause outbreaks of febrile illnesses and hemorrhagic fevers [1,2]. These viruses have established urban cycles, which have led to severe public health issues. Additionally, they are continuously spread and maintained in nature through sylvatic cycles involving various vertebrate animals, such as rodents, birds, and non-human primates (NHPs), as well as mosquito vectors, including *Aedes aegypti*, *Haemagogus* sp., *Sabethes* sp., and *Culex* sp. [3,4].

Zoonotic viruses of public human health interest have been reported in NHPs, and similar pathogens (e.g., viruses that cause Ebola, HIV/AIDS, and malaria, among others) can infect both humans and NHPs [5–9]. In Brazil, arboviruses such as the *Orthoflavivirus flavi* (YFV) and *Orthoflavivirus zikaense* (ZIKV), *Orthoflavivirus denguei* (DENV), the alphaviruses Mayaro virus (MAYV) and Chikungunya virus (CHIKV), and the orthobunyavirus Oropouche virus (OROV) have been identified in NHPs [10,11]. Moreover, YFV has undergone spillover and adapted to a sylvatic cycle involving Neotropical NHPs (*Cebidae, Aotidae*, and *Callitrichidae*). From 2015 to 2019, Brazil recorded almost 1000 human deaths and over 15,000 epizootic cases in NHPs [12,13], with howler monkeys (*Alouatta* sp.) being the most affected genus [14]. Recent studies have suggested the role of *Callithrix* sp. in maintaining urban YFV [15]. In 2013–2014, ZIKV emerged in Brazil [15], and the first cases of ZIKV infecting domestic and free-ranging Neotropical NHPs were reported in *Callithrix jacchus* and *Sapajus libidinosus* [16]. In 2018, genomic evidence of ZIKV was found in *Callithrix* sp. and *Sapajus* sp., suggesting that a sylvatic cycle had been established [16]. Other emergent orthoflaviviruses, such as SLEV, have been identified and likely originated in Central American vectors [17]. It was first described in an outbreak in the USA in 1933 [18]. The virus was first described in Brazil in 1960 in the vector *Culex declarator* [19] and later identified in rodents, birds, and NHPs in the Amazon basin [20,21]. SLEV was isolated from two human cases in the Amazon basin in the 1970s [21], and again in the early 2000s, when it was isolated from a human case during a dengue outbreak in São Paulo State [22]. More recently, SLEV was described during ZIKV and DENV outbreaks [23] in the Ribeirão Preto region of northeastern São Paulo State, alongside a large dengue outbreak, and among humans and equids in the southeast region of Brazil [24]. The virus was commonly involved in enzootic cycles, including *Culex* mosquitoes and birds as amplifying hosts [19]. Moreover, SLEV is identified in horses from different regions of Brazil [25–28]. Recent serological evidence of SLEV among NHPs has been reported for *Atelidae*, *Callitrichidae*, and *Cebidae* in different regions of the country [29], underlying SLEV complex epidemiology in Neotropical primates. Other orthoflaviviruses include Rocio virus (ROCV) and *Orthoflavivirus ilheusense* (ILHV), which are responsible for sporadic human cases and have been identified in birds, sloths, rodents, and NHPs [30–32].

Eco-surveillance of arboviruses in NHPs is necessary to avoid human outbreaks and to monitor and protect threatened NHP populations. Studying the ecology and evolution of these viruses is imperative to understanding how they are transmitted and maintained in NHP populations. Epizootics in NHPs function as sentinel events for detecting human cases. By identifying disease events that occur simultaneously in several NHPs from the same geographical area, we can understand whether they have the potential for spillover events in humans. As part of these studies, risk maps are useful tools to identify areas of higher density of viral infections in animal populations (hotspots) and a suitable strategy to predict the onset of human cases. Therefore, here we have evaluated active infection with arboviruses in blood, saliva, feces, and urine samples obtained from free-range or captive NHPs or NHP carcasses. We have also assessed the ecological patterns related to infection with arboviruses in NHPs and identified hotspot areas in southeastern Brazil.

## Methods

### Ethics statement

All the NHPs were handled according to the guidelines of the American Society of Mammalogists [33]. Procedures followed wildlife protection laws, under license n.19838-6. The project was approved by the Animal Research Ethics Committee of the University of São Paulo (No. 020/2011), following the Brazilian Ethics Standards.

### Study area and sampling

**Fig 1:**
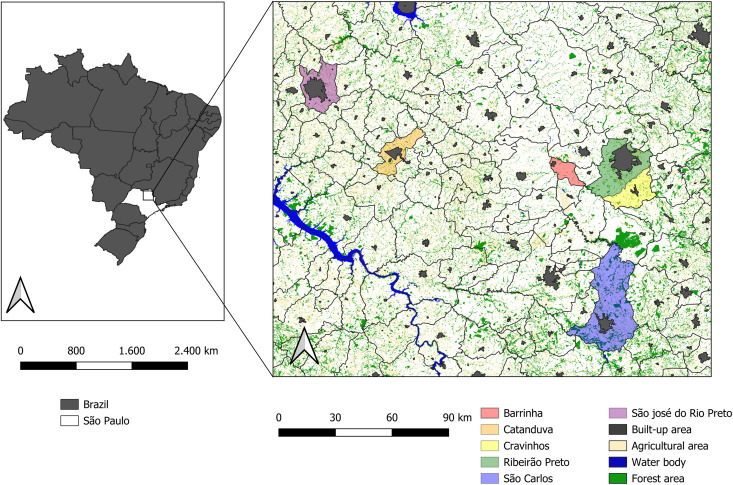
Study area, sampling points in southeastern Brazil. The map highlights the six sampling sites in the northeast of São Paulo State. Areas with native vegetation of Cerrado patches and Atlantic Forest enclave are represented in green. In yellow are agricultural areas, and in grey is urbanization. The map was generated by QGIS 3.26.0 (Zürich-CH). The base layer used in this map are from Instituto Brasileiro de Geografia e Estatística (IBGE) https://www.ibge.gov.br/geociencias/downloads-geociencias.html.

We sampled a total of 301 NHPs, including 56 captive and 245 free-range individuals, between 2017 and 2020. The NHPs, both captive and free-range, were captured in forest patches and urban areas in the northern region of São Paulo State, southeastern Brazil (Fig 1). Captive NHPs were sampled in zoos located in the counties of Catanduva, São Carlos, São José do Rio Preto, and Ribeirão Preto in São Paulo State. Biological materials of deceased NHPs were collected in partnership with the Health Surveillance Department (HSD) of the county of Ribeirão Preto. Samples included oral and rectal swabs, feces, salivary glands, brain tissues, liver tissues, and urine. Several carcasses collected by the HSD were unsuitable for preservation, hindering accurate species identification.

Free-range NHPs were sampled in forest patches in the counties of Barrinha, Catanduva, and Cravinhos (Fig 1) between April 2017 and October 2018, which covered dry (April to September) and rainy (October to March) seasons, to identify the NHP prevalence per season. The predominant landscape in the studied areas is the Cerrado (savanna-like vegetation), interspersed with patches of Atlantic Forest and monocultures (e.g., sugarcane, grassland) along the human-animal interface.

Tomahawk traps of three sizes (90 cm x 40 cm x 40 cm, 70 cm x 40 cm x 40 cm, or 45 cm x 21 cm x 21 cm) were placed 20 meters apart along a one-kilometer transect. At each station, five traps were positioned in the canopy. The traps were baited with a mixture of banana, rolled oats, sardines, vanilla essence, and ground peanut candy for four to six days and set for three consecutive nights. The captured NHPs were anesthetized with a combination of ketamine and midazolam; anesthetic dosage depended on the species’ body mass. The protocols followed the guidelines of the Animal Research Ethics Committee of the University of São Paulo, in accordance with the Brazilian Ethics Standards. The NHPs were monitored for complete post-anesthesia recovery and returned to the capture sites on the same day. NHP species were identified based on standard taxonomic keys [34–36].

### Arbovirus detection

We collected 1169 samples from 301 NHPs, including 225 blood samples, 289 rectal swabs, 95 oral swabs, 206 liver tissue samples, 209 salivary glands, 137 brain tissue samples, and 8 urine samples. Of these, 509 samples from 248 NHPs were analyzed using semi-nested multiplex RT-PCR for the *Orthoflavivirus* NS5 gene [36] (Table 1). To detect members of the genus *Alphavirus*, 203 blood samples were analyzed by RT-qPCR for genes NS1 of MAYV [37] and CHIKV [38] (S1 Table). Autopsy reports of the infected NHPs were analyzed to identify signs of infections or sickness (S2 Table). To mitigate the risk of contamination during the assays, well-characterized standard viral strains were employed as positive controls and included MAYV BeAr20290, CHIKV S27 – African, DENV-1 Mochizuki, DENV-2 NGC 84850, DENV-3 H87, DENV-4 H241, SLEV BeH 355964, ILHV BeH7445, ROCV SpH 34675, West Nile NY99, ZIKV SpH 2015, and YFV BeAn 131. All viral strains were sourced from the biorepository of samples and viruses at the Center for Virology Research, Ribeirão Preto Medical School, University of São Paulo, and have not been obtained commercially.

**Table 1:** Sample number of *Orthoflavivirus* (RT-PCR) and *Alphavirus* (RT-PCR and serology) in collected NHP.

	Nº NHP samples	Collected samples							Total
		Blood	Liver	Salivary gland	Oral swab	Brain	Rectal swab	Urine	
	301	225	206	209	95	137	289	8	1169
RT-PCR *Flavivírus*	248	225	28	28	47	9	164	8	509
qPCR *Alphavírus*	203	203	–	–	–	–	–	–	203
ELISA *Alphavírus*	209	209	–	–	–	–	–	–	209
Eco-epidemiological Analysis	194	–	–	–	–	–	–	–	194

### CHIKV and MAYV ELISAs

A total of 209 blood samples obtained from NHPs were analyzed using in-house CHIKV and MAYV ELISAs targeting viral recombinant E2 proteins (rE2) as antigens described by Fumagalli [39,40] (S1 Table). A peroxidase conjugate anti-Monkey IgG (Sigma) from our laboratory antibody library was employed in both tests.

### Virus isolation

Attempts to isolate arboviruses were made in samples that showed *Orthoflavivirus* amplicons in the semi-nested multiplex RT-PCR. The samples were filtered through 0.22-μm pores to eliminate bacteria. Then, 200 μL of the sample was inoculated into *Aedes albopictus* cells (C6/36 - CRL-1660) monolayers, in three blind passages, to increase viral titer, as previously described [41]. Viral isolation was confirmed by semi-nested multiplex RT-PCR for the *Orthoflavivirus* gene NS5 (S1 Table) [36]. Viral isolation was further confirmed by indirect immunofluorescence assay (IFA). At five or six days post-infection, infected cells were transferred onto slides and incubated with murine ascitic fluid containing polyclonal antibodies specific to each *Orthoflavivirus* species (laboratory stock), followed by incubation with an Alexa Fluor-conjugated secondary antibody against mouse immunoglobulins. The presence of viral particles was confirmed by the detection of specific fluorescent signals under a confocal microscope.

### Nucleotide sequencing

*Orthoflavivirus* amplicons in semi-nested multiplex RT-PCR were sequenced by the Sanger method in a Genetic Analyzer 3500 (Applied Biosystems); previously described primers were used (S1 Table) [36–38].

### Sequence annotations

Fragments of the NS5 segments were generated in this study and were deposited in GenBank under the accession numbers: OQ290696, OQ290697, OQ290698, OQ290699, OQ290700, OQ290701, OQ290702, OQ290703, OQ290704, OQ290705, for SLEV and OQ032518 for DENV-3.

### Sequence analysis

For sequence analysis, the Dengue Virus Typing tool (Genome Detective Tool, Version 4.1) was used to genotype the DENV-3 genomic fragment based on genotype and major lineage (S1 Fig, S2 Fig). Representative sequences from within genotype III of DENV-3 were selected for analysis, with the DENV-3 reference sequence (GenBank accession number: OK469356) used as an outgroup. Additionally, were added three representative sequences with identity ranging from 99.1 to 98.2%, namely GU131862 (Ribeirão Preto-SP-BR), OQ727062 (Manaus-AM-BR) and KC425219 (Pernambuco-AL-BR) The MAFFT v7.490 tool was employed to align the sequences [42]. A maximum likelihood phylogenetic tree was generated with the IQ-TREE software (version 2.2.0). A substitution model was automatically chosen by using the ModelFinder module [43], and for the branch test, we used SH-aLRT [44], both present in IQ-TREE2 [45]. For SLEV sequencing, the NS5 fragments were aligned to the EF158049.1 sequence using MAFFT-linsi-add fragments. Alignment graphic representations were made by Geneious 2024.02 (www.geneious.com).

### Data analysis

Seasonality, sex, age classes, biometric measures, and presence of wounds were analyzed to identify ecological patterns related to infections with arboviruses. Cluster analyses were performed to group the biometric data of body mass, body measurements, tail, forearm, hindfoot, and ear of each NHP. The Euclidean distance and dissimilarity measure were applied to define the number of clusters (by using the sum of intra-cluster squares), adjusted by the non-hierarchical “k-means” method. “Cluster 1” and “Cluster 2” grouped NHPs with highly similar biometric measurements and low biometric measurements, respectively. The mean, standard deviation, and median were calculated from the biometric data. The relationship between variables and viral infection prevalence ratios (PR) was analyzed by using simple and multiple Poisson regression models [46]. Please see raw data in supporting information: S5 Table Ecological and virological data of NHP. All the figures were made using the R program (version 4.0.4), and analyses were performed by employing SAS 9.4. A significance level of 5% was adopted for all the analyses [47]. The QGIS 3.26.0 program was used to create distribution and risk maps. Influence radius and Kernel density functions were determined for the risk map in locations with a higher number of analyzed NHPs.

## Results

### NHP species diversity

We studied 301 non-human primates (NHPs), including both captive and free-range individuals, in the north of São Paulo State, from April 2017 to October 2020. Of the 245 free-range NHPs, 211 (85.1%) were *Callithrix* sp., 13 (5.2%) were *Alouatta* sp., 19 (7.7%) were *Sapajus* sp., 4 (1.6%) were *Saguinus midas*, and 1 (0.4%) was *Saimiri sciureus* (Table 2). We collected samples from NHPs in Ribeirão Preto (n = 256), Catanduva (n = 16), Cravinhos (n = 6), Barrinha (n = 9), São Carlos (n = 7), and São José do Rio Preto (n = 7) (Fig 1). Captures were conducted in areas characterized by two distinct forest landscapes: Atlantic Forest enclaves interspersed with patches of Cerrado (scrub savanna) and along the edges between natural forest and monoculture crops (soybean, grassland, corn, and coffee) (Fig 1). Male and female NHPs did not differ in the number of animals infected with arboviruses (*p* = 0.18). Adults were more prevalent during the dry season (n = 141) compared to the rainy season (n = 53), regardless of NHP species (*p* = 0.001) (Table 2).

**Table 2:** Distribution of tested animals according to sex, age classes, and seasonality.

	Dry Season (April to September) Abundance	Rainy Season (October to March) Abundance		
	No. (%)	No. (%)	Total No. (%)	*p* value
Sex				
Males	75 (66.4)	38 (33.6)	113 (100)	0.18
Females	78 (75)	26 (25)	104 (100)	
Age*				
Young	2 (16.7)	10 (83.3)	12 (100)	
Subadults	10 (90.90	1 (9.10	11 (100)	0.001
Adults	141(72.7)	53 (27.3)	194 (100)	

* Age according to the specific body mass for each species and secondary external sexual features.

### *Orthoflavivirus* diagnosis by RT-PCR

Of the 301 NHPs collected, 248 had samples that could be tested. Among the 248 NHPs tested, 30 (12%) were found positive for orthoflavivirus infection (Table 3). Each NHP provided blood samples, oral swabs or salivary glands, rectal swabs, liver fragments, brain tissue, and occasionally urine. Therefore, when available, each collected NHP generated up to 7 samples for analysis (Table 1). Forty-nine samples (blood samples (29), oral swabs (8), salivary glands (1), rectal swabs (10), and urine samples (1)) were positive for *Orthoflavivirus* amplicons. Positive samples were identified in *Sapajus apella* (DENV-1, DENV-2, and SLEV), *Callithrix penicillata* (DENV-1, DENV-3, SLEV, YFV, and ZIKV), *Saguinus midas* (SLEV), and *Alouatta caraya* (SLEV and ZIKV) (Table 4). In addition, two *Alouatta caraya* specimens were coinfected with ZIKV and SLEV, and one *Callithrix penicillata* specimen was coinfected with ZIKV and DENV-3. We detected seven distinct *Orthoflavivirus* amplifications by RT-PCR.

**Table 3:** NHP species tested for *Orthoflavivirus* and *Alphavirus,* and distribution according to sex and age classes.

Gender	Species	N (%)	Male	Female	Sex ratio	Young	Subadult	Adult	*Orthoflavivirus* positivity (%)
*Callithrix*	*penicillata*	209 (84.3)	106	103	0.53	20	9	180	18/209 (8.6)
	*jacchus*	1 (0.4)	0	1	–	0	0	1	0
	*geoffroyi*	1 (0.4)	1	0	–	0	0	1	0
*Alouatta*	*caraya*	12 (4.8)	4	8	0.33	2	2	8	4/12 (33.3)
	*guariba*	1 (0.4)	1	0	–	0	0	1	0
*Saguinu*	*midas*	4 (1.6)	1	3	0.25	0	0	4	2/4 (50)
*Saimiri*	*sciureus*	1 (0.4)	1	0	–	0	0	1	0
*Sapajus*	*apella*	10 (4.0)	3	7	0.3	0	0	10	6/19 (31.6)
	*libidinosus*	7 (2.8)	1	6	0.14	2	2	3	0
	sp*	2 (0.8)	2	0	–	0	0	2	0
Total		248	120	128	0.51	24	13	211	30/248 (12.1)

* Not possible to identify the species level

**Table 4:** Orthoflaviviruses identified by amplicon according to the NHP species, including data on viral isolation.

*Orthoflavivirus* Identified	Number of infected NHP	Species of NHP	Prevalence of *Orthoflavivirus* per species of infected NHP (%)	Positive Samples	Isolated Samples	Sequenced Samples
DENV 1	1	*Callithrix penicillata*	1/209 (0.5%)	B	**–**	
	1	*Sapajus apella*	1/10 (10%)	B	B	
DENV 2	1	*Sapajus apella*	1/10 (10%)	B	–	
DENV 3	2	*Callithrix penicillata*	2/209 (1%)	B	–	B
YFV	1	*Callithrix penicillata*	1/209 (0.5%)	B	–	
SLEV	3	*Alouatta caraya*	3/12 (25%)	B, SG, OS, RS	B, OS, RS	B, OS
	7	*Callithrix penicillata*	7/209 (3.4)	B, OS, RS	B, OS, RS	B, OS, RS
	2	*Saguinus midas*	2/4 (50%)	B, OS	OS	OS
	4	*Sapajus apella*	4/209 (1.9%)	B, OS, RS, U	OS, RS	B, OS, U
ZIKV	3	*Alouatta caraya*	3/12 (25%)	OS, SG, RS	OS	
	8	*Callithrix penicillata*	8/209 (3.8%)	B	B	

DENV = *Orthoflavivirus denguei*, SLEV = *Orthoflavivirus louisense*, ZIKV = *Orthoflavivirus zikaense*, YFV = *Orthoflavivirus flavi*, OS = oral swab, RS = rectal swab, SG = salivary gland, B = blood, U = urine

### Serological and molecular screening for alphaviruses

We tested sera collected from 209 NHPs by two IgG rE2 ELISAs, which provided negative results for both MAYV and CHIKV (S3 Fig). Furthermore, blood samples collected from 203 NHPs were negative for alphaviruses, as revealed by RT-qPCR. We did not detect MAYV or CHIKV genomic fragments.

### Virus isolation

**Fig 2:**
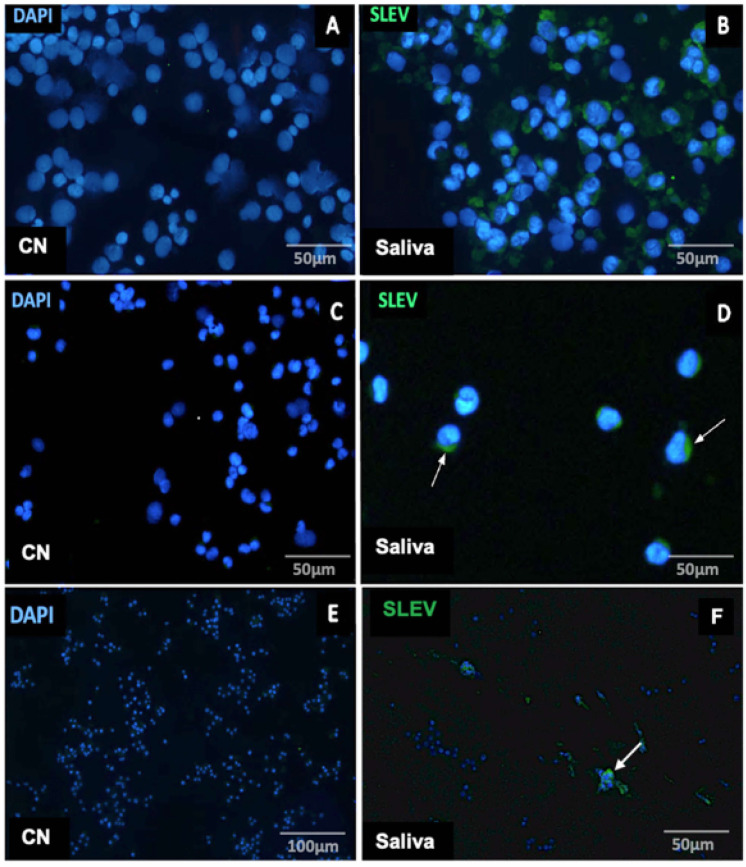
Saliva from non-human primates contains infectious *Orthoflavivirus louisense* (SLEV) detectable by indirect immunofluorescence assay (IFA) in VERO and C6/36 cells. Viral protein labeling (green) was detected using polyclonal antibodies against SLEV, and nuclei were counterstained with DAPI (blue). Panels A and C show uninfected VERO cell controls (CN). Panels B and D display VERO cells infected with SLEV-positive saliva samples from *Saguinus midas* (B) and *Alouatta caraya* (D), respectively. Panel E shows uninfected C6/36 cell controls (CN), and panel F shows C6/36 cells infected with SLEV-positive saliva from *Sapajus apella*. White arrows indicate areas of viral protein labeling (green). Cells were fixed and processed for IFA five to six days post-infection. Scale bars: 50 µm (A-D, F) and 100 µm (E).

Three viruses, DENV-1, SLEV, and ZIKV, were successfully isolated in C6/36 and VERO cells (Table 4) from 12 samples after three consecutive blind passages. All viral isolations were confirmed by semi-nested multiplex RT-PCR. Notably, SLEV was isolated from the saliva of different NHP species, and its presence was further confirmed by indirect immunofluorescence assay (IFA) (Fig 2).

### *Orthoflavivirus* NS5 nucleotide sequences

We sequenced all the *Orthoflavivirus* amplicons obtained by semi-nested multiplex RT-PCR by the Sanger method, using specific primers [37]. We determined *Orthoflavivirus* NS5 nucleotide sequences in 11 amplicons obtained from 11 NHPs (S3 Table).

**Fig 3:**
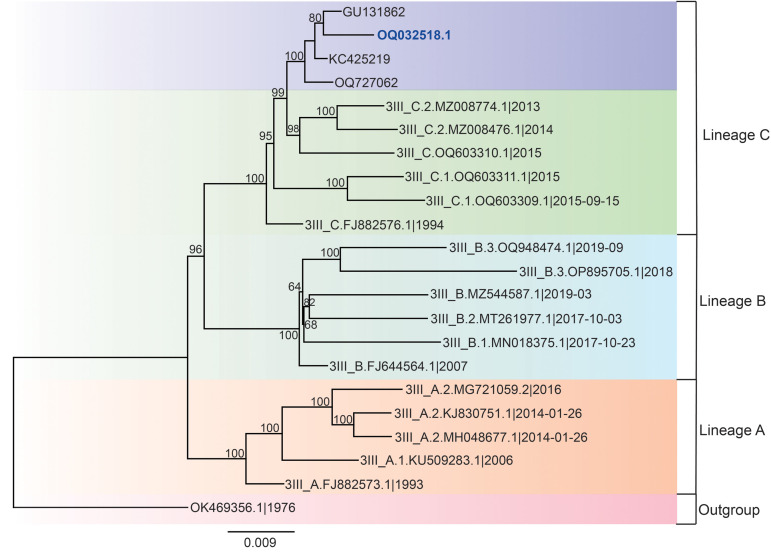
Phylogenetic analysis reveals that the isolated *Orthoflavivirus denguei* is closely related to *Orthoflavivirus denguei* 3 (DENV-3) genotype III, major lineage C. Maximum-likelihood phylogenetic tree of DENV-3 based on the NS5 gene segment. SH-aLRT support values are indicated at the branch nodes. The tree was inferred using the HKY85 + F nucleotide substitution model. Sequences in the branch highlighted in dark blue represent Brazilian DENV-3 isolates. In contrast, the sequence obtained in this study (GenBank accession no. OQ032518.1) corresponds to DENV-3 genotype III, major lineage C, and is highlighted in blue. The sequence OK469356.1 was used as an outgroup reference.

We constructed a phylogenetic tree by using complete or partial sequences of DENV-3. Our DENV-3 genomic fragment clustered with samples previously isolated from DENV patients in southeastern Brazil, namely in Ribeirão Preto and other sites in Pernambuco and Manaus (Fig 3). Additionally, we classified our fragment as genotype III (S1 Fig) and major lineage C (S2 Fig). Regarding the 10 SLEV genomic fragments, although their small length (ranging from 23 bp to 173 bp) (S3 Table), phylogenetic analysis of the ten nucleotide sequences obtained was attempted; however, the short sequence lengths precluded sufficient data for inferring their contribution to SLEV epidemiology in Brazil (S4 Fig). A comparison of the alignment of these 10 samples revealed high homology between the fragments (S4 Fig). No amino acid residue variations were detected in any of the partial gene sequences obtained in our study, and all nucleotide variations were classified as synonymous mutations. However, the genomic fragments of sequences OQ290702 and OQ290699 exhibited a cytosine at position 8,427, instead of the thymine observed in the reference sequence and other genomic fragments. Moreover, sequence OQ290699 displayed four additional nucleotide variations: a thymine instead of a cytosine at position 8,367; a guanine instead of an adenine at position 8,379; and adenine instead of guanine at positions 8,382 and 8,412 (S4 Fig).

### Eco-epidemiology and risk map of NHPs infected with orthoflaviviruses

**Fig 4:**
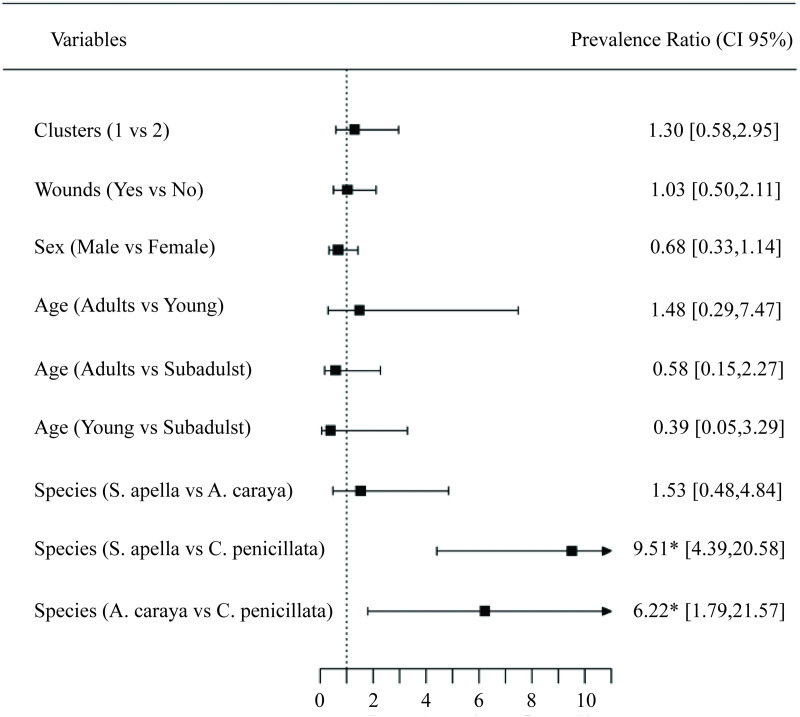
Eco-epidemiological species-related differences of *Orthoflavivirus* infection prevalence among non-human primates in southeastern Brazil. Prevalence ratios (95% confidence intervals) for *Orthoflavivirus* infection were estimated based on morphometric, behavioral, sex, age, and species characteristics of non-human primates (NHPs). Variables included allometric cluster (Cluster 1 = animals with higher allometric measurements; Cluster 2 = animals with lower measurements), presence of wounds (Yes/No), sex, age category, and species (*Sapajus apella*, *Alouatta caraya*, and *Callithrix penicillata*). A significantly higher infection prevalence was observed in *S. apella* and *A. caraya* compared with *C. penicillata* (*p* < 0.01). CI = confidence interval; vs = versus.

The following NHPs were infected with orthoflaviviruses: *Saguinus midas* (2, 50% of the *Saguinus midas* investigated here), *Sapajus apella* (6, 31.6% of the investigated *Sapajus apella* investigated), *Alouatta caraya* (4, 33.3% of the investigated *Alouatta caraya*), and *Callithrix* *penicillata* (18, 8.6% of the investigated *Callithrix penicillata*) (Table 3). Infection with orthoflaviviruses was not significantly associated with biometric data, age classes, wounds, or sex (Fig 4). However, there was a significant association between NHP species and the prevalence of infection. When comparing different species of infected animals, it is evident that NHPs of the species *Sapajus apella* had a 90.49% higher incidence of orthoflavivirus infection than *Callithrix penicillata* (*p* < 0.01) (Fig 4). Similarly, individuals of the species *Alouatta caraya* exhibited a 93.78% higher prevalence of orthoflavivirus infection compared to *Callithrix penicillata* (*p* < 0.01) (Fig 4).

**Fig 5:**
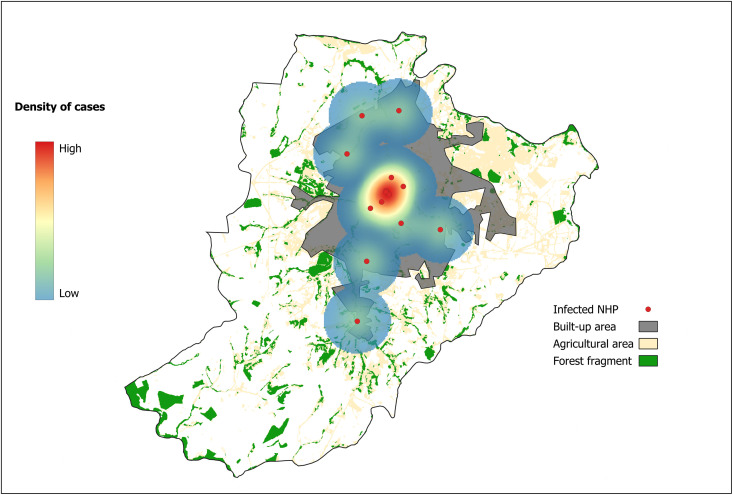
Spatial distribution and density of *Orthoflavivirus*-infected non-human primates (NHPs) in Ribeirão Preto County, São Paulo State, Brazil. Kernel density estimation (KDE) risk map showing the spatial distribution and density of *Orthoflavivirus*-infected NHPs (red circles) in Ribeirão Preto County. Mapping and spatial analyses were performed in QGIS version 3.26.0 using distance matrices between confirmed arbovirus cases. The quartic kernel function was applied, providing higher weighting to nearby points and lower weighting to distant ones, with bandwidth values of 2,562.09 for the complete dataset and 3,106.44 when considering only the genus of interest. A continuous KDE classification was employed for improved visual representation. Built-up areas, agricultural zones, and forest fragments are indicated in grey, beige, and green, respectively. Base layers were obtained from the *Instituto Brasileiro de Geografia e Estatística* (IBGE; https://www.ibge.gov.br/geociencias/downloads-geociencias.html).

Among the studied areas, the county of Ribeirão Preto concentrated the highest density of sampled NHPs infected with orthoflaviviruses (14) (S4 Table), so we selected it to construct a Kernel density risk map of infected NHPs. The map highlights the incidence of infected NHPs at the borders and other regions of the county (Fig 5). Infected NHPs were found mainly in parks or urban green areas of the county, and most cases were concentrated near the zoo ecological park. When compared to human cases reported in the study areas during the same period, we observed an increase in dengue cases, particularly in Catanduva, in contrast to Ribeirão Preto (S5 Fig). However, an inverse trend was observed for Zika virus (ZIKV) (S6 Fig) and Chikungunya virus (CHIKV) (S7 Fig) cases, as reported by the Health Surveillance Departments (HSDs) of the respective cities. The HSDs of the other municipalities included in our study did not provide data on the incidence of ZIKV, CHIKV, or DENV cases.

## Discussion

In this study, we found evidence of infections with orthoflaviviruses of interest to public health among evaluated species of NHPs. The prevalence of these infections was 12% in urbanized areas, which highlighted hotspots of arboviruses in southeastern Brazil. For the first time, we have detected infection with SLEV in the golden-handed tamarin (*Saguinus midas*), a previously undocumented orthoflavivirus in this NHP, and we also identified coinfections with ZIKV and DENV-3 and with ZIKV and SLEV in the black-tufted marmoset (*Callithrix penicillata*) and the black howler monkey (*Alouatta caraya*), respectively. We detected SLEV and ZIKV in saliva samples and rectal swabs collected from NHPs, a potential route for non-vector transmission of the virus, which needs to be proven in future studies. This is relevant because ZIKV, DENV, SLEV and YFV have the potential to cause widespread human epidemics.

Analysis of serological and molecular data has suggested that Neotropical wild mammals such as rodents, marsupials, and bats maintain orthoflaviviruses, including DENV, in nature [48]. However, the role of Neotropical primates in the epidemiology and transmission of arboviruses has not been described yet, despite evidence that NHPs are susceptible to alphavirus and orthoflavivirus infections [4,49,50]. In our study, we found 33 genomic fragments of *Orthoflavivirus* in 30 NHPs, including SLEV, ZIKV, DENV-1, DENV-3, DENV-2, and YFV. Among the infected NHPs, *Callithrix penicillata* was the most common, followed by *Sapajus apella*, *Alouatta caraya*, and *Saguinus midas*. The larger number of infected *Callithrix* sp. is associated with the larger number of samples collected from this species, so we cannot affirm that differences among the species of NHPs are significantly associated with disease dynamics or susceptibility due to this trap bias. *Callithrix* sp. have been introduced more recently (20–30 years ago) into the region and are widely distributed in the Atlantic Forest and Cerrado biomes, occupying niches previously held by other natural species [51]. In addition, *Callithrix* sp. are highly adapted to urbanized areas and forest fragments, which are both present in the study region [34,51]. Moreover, all the studied species inhabit urban forests, thereby increasing their potential as sentinels and/or hosts within the zoonotic cycle and raising the concern that they participate in spillover events.

We found that five NHPs were infected with DENV. More specifically, *Callithrix penicillata* and *Sapajus apella* were infected with DENV-1, *Sapajus apella* was infected with DENV-2, and *Callithrix penicillata* was infected with DENV-3. The clinical presentation of dengue infections in NHPs is poorly understood. We found impaired internal organs based on the necropsy report of a *Callithrix penicillata* infected with DENV-1. However, further studies on NHPs infected with DENV are necessary to understand and characterize the disease in these NHPs. The infected NHPs in our study were found mostly in green urban areas, hence their limited movement could facilitate a local spillback event, supported by vectors such as *Aedes* sp. or *Culex* sp. According to the Health Surveillance Division (DVS) of the city of Ribeirão Preto and Catanduva, dengue cases showed a significant increase during the period of this study (2017–2020) [52]. However, ZIKV and CHIKV cases monitored by the DVS declined during the same period in Ribeirão Preto city and were not reported in Catanduva city. These trends are consistent with our findings, suggesting that the cases of DENV-infected NHPs observed in our study reflect the dynamics of human cases during the outbreaks. Moreover, this association is supported by our phylogenetic findings. A well-supported cluster between our DENV-3 sequence and a previous human sequence from the same region suggests that the virus may still be silently circulating in NHPs. This raises concerns about the need for proper sylvatic surveillance to prevent potential spillover events. Sylvatic cycles of DENV involving NHPs have been described in Africa and Asia and have been suggested in the Americas, particularly in Panama, Puerto Rico, and Argentina [53]. However, there is limited evidence of NHPs infected with DENV in South America [54], including Brazil [55–57], and evidence suggests a spillback event rather than a sustained sylvatic cycle [56,58]. Nevertheless, most studies have presented serological findings, which are prone to cross-reactivity among other orthoflaviviruses [59]. On the other hand, our findings suggest that the virus or its genomic material is present, which could indicate active infection rather than prior exposure alone. While there is limited evidence of a sylvatic cycle of DENV in the Americas [3], our findings contribute to the accumulating evidence of a sylvatic cycle of DENV in southeastern Brazil.

ZIKV is an important arbovirus in Brazil; it has caused large outbreaks and hundreds of cases of congenital disease with microcephaly [31]. Here, we identified ZIKV in *Alouatta caraya* and *Callithrix penicillata*. We successfully isolated ZIKV from NHP saliva. Despite the established evidence of non-vectorial transmission of ZIKV through saliva [46,47] and during sexual relations [47] in humans, the dynamics of ZIKV transmission among NHPs have not been fully characterized. Nonetheless, our findings suggest ZIKV maintenance among NHPs. However, further investigations employing a larger sample size are needed to confirm this observation and to characterize the ZIKV transmission pathway in NHPs.

We observed infection with SLEV in all the NHP species except for *Saimiri sciureus*. We found SLEV in blood samples, saliva samples, salivary glands, urine samples, and rectal swabs collected from the NHPs. Although SLEV is considered an ornithophilic orthoflavivirus, several studies have suggested various hosts, including mammals such as rodents and NHPs [20,60–62]. Indeed, infection of *Alouatta caraya*, *Sapajus nigritus*, *Sapajus cay*, *Leontopithecus chrysomelas*, and different *Sapajus* sp. with SLEV has been shown in the northern and northeastern regions of Brazil [29,63]. Although our study supports the evidence that mammals, such as NHPs, could be involved in the sylvatic or transmission cycle of SLEV in nature, it is important to highlight that we describe the genomic material of SLEV, which may indicate an ongoing infection. This finding is highly significant for public health surveillance. Despite the limitations of our sequencing approach concerning fragment size, the data suggest that fragments from NHPs collected at the same site exhibited higher homology among each other compared to those from NHPs collected at different sites. This scenario suggests that, even when targeting a highly conserved *Orthoflavivirus* protein such as NS5, our sequencing approach was able to generate data indicating that NHPs from the same location likely shared a common ancestral virus and were infected by the same viral strain. In contrast, the NHPs infected at different sites may have been exposed to more diverse strains. However, further evidence is needed to confirm this hypothesis.

Curiously, we found that a *Callithrix penicillata* was coinfected with ZIKV and DENV-3 and that an *Alouatta caraya* was coinfected with ZIKV and SLEV, which highlights the abundance of orthoflaviviruses circulating in the region. Even though human disease by SLEV is sporadic in Brazil [64], coinfections with SLEV and DENV have been reported. SLEV in a patient with neurological symptoms was diagnosed in the middle of a large DENV-3 outbreak in the north of São Paulo State [65]. SLEV might be silently circulating in sylvatic cycles, including NHPs, in the region, so continued eco-surveillance is needed to substantiate this assertion.

We evaluated ecological patterns such as sex, age classes, biometric features, and environmental factors associated with infections with arboviruses in NHPs and found that they were not determinant. Hence, NHPs of any age and sex and in any season are expected to be exposed to mosquito bites and could become infected [14]. Body mass, a biometric feature, was not associated with *Orthoflavivirus* infection. This contrasts with previous studies suggesting that animals with smaller body mass, shorter life spans, and low reproductive rates are more susceptible to viral infection and persistence [65]. Furthermore, other authors have suggested that the higher reproduction rate of NHPs favors the establishment of a sylvatic cycle of ZIKV [66].

As previously described, NHPs and other mammals are implicated in the sylvatic transmission of the alphaviruses MAYV and CHIKV in the Americas [50]. However, we did not detect MAYV or CHIKV infection during serology and RT-qPCR of the NHP samples. MAYV and CHIKV are known to circulate in endemic areas in north and northeastern Brazil and were found during the DENV, YFV, and ZIKV outbreaks [67,68]. MAYV has been reported to have caused human infections in the county of São Carlos, in São Paulo State [69]. Notably, misdiagnosis of both CHIKV and MAYV acute infections as dengue and other causes of acute febrile illnesses is highly probable [69]. Additionally, 1% of all dengue-like febrile illnesses in northern South America are estimated to be caused by MAYV [70]. This result underscores the necessity of eco-surveillance expansion to comprise other neglected arboviruses of human health importance, especially during seasonal outbreaks.

*Callithrix* sp. have been largely investigated for arboviruses, and there is strong evidence that these NHPs can participate in the transmission and maintenance of orthobunyaviruses like Oropouche virus (OROV) [71] and orthoflaviviruses such as YFV [72] and ZIKV [73]. In our study, *Callithrix* sp. were trapped in urban areas or alongside peri-urban regions (the human-animal interface), where this primate overrepresented the collected NHPs. Our risk map shows species of NHPs infected with orthoflaviviruses in the county of Ribeirão Preto, and *Callithrix penicillata* accounted for most of the studied NHPs. The map shows that infected animals are scattered throughout the county, with greater density around downtown areas, particularly in the surroundings of the zoo ecological park. This area is forested, and wild NHPs are frequently observed there. In the zoo, the captive animals probably create a wildlife environment that allows arboviruses to circulate, hence the highest incidence of infected NHPs. Therefore, our findings recommend surveillance of NHPs and other mammals in the zoo. Hotspots of infections with arboviruses are important warnings for implementing control, prevention, and remediation measures regarding future outbreaks. Well-succussed surveillance results were achieved for YFV in the south of Brazil in 2008–2009 and in São Paulo State in 2017–2018, as revealed by the reports of the deceased NHPs. In both cases, finding deceased primates led to extensive vaccination of human populations, avoiding outbreaks [74,75]. Here, we found that one *Callithrix penicillata* was infected with YFV during the yellow fever outbreak from 2016 to 2019. This is supported by previous studies showing that *Callithrix* marmosets had high positivity rates for YFV when compared to other species [10]. Our results show that YFV circulates in a sylvatic cycle in the investigated region, raising concern about the emergence of human cases. Indeed, a new highly infective strain of YFV (Clade 1E) was found to circulate in São Paulo State during the 2016–2019 outbreak [76].

*Sapajus* sp. and *Alouatta* sp. are both considered medium to large-sized Neotropical NHPs, compared to *Callithrix* sp. Even though body mass was not significantly related to orthoflaviviruses infections, our results challenge studies supporting smaller Neotropical NHPs as more susceptible to orthoflavivirus infections, such as ZIKV [66]. It should be considered that the low diversity of NHPs collected in our study is due to trap bias. Therefore, the high infection rates among *Sapajus* sp. and *Alouatta* sp become prominent, compared to smaller NHPs collected in our study. Moreover, it is important to consider the sampling bias that may have influenced this observation. The most infected species of *Sapajus* sp. and *Alouatta* sp. shared the same region and enclosure environment. This scenario could have facilitated the virus transmission. This result shows *Sapajus* sp. and *Alouatta* sp. are highly susceptible to orthoflavivirus infection, and their social behavior yields an intergroup virus transmission with no apparent signs of infection.

The present study provides evidence that emerging orthoflaviviruses (SLEV, DENV 1–3, YFV, and ZIKV) cocirculate among NHPs in the north of São Paulo State, Brazil. To the best of our knowledge, this is the first report of infection with SLEV in the golden-handed tamarin *Saguinus midas* and of coinfections with ZIKV and DENV-3 and with ZIKV and SLEV in the black-tufted marmoset (*C. penicillata*) and the howler monkey (*Alouatta caraya*), respectively. Our risk map shows hotspots for the potential spread of DENV, SLEV, and ZIKV in the county of Ribeirão Preto. In short, our data provide support for public health policies and encourage continuous eco-epidemiological surveillance of arboviruses among free-range and captive NHPs and other wildlife in tropical and subtropical countries.

## Supporting information

S1 Table Sequence of primers for RT-PCR, RT-qPCR and the ELISA antigens used in our study. M-N = Multi nested, N**(-) = Nested primer, (+) = Forward primer, (-) = Reverse primer, NS = Non structural protein, r = Recombinant antigen, NP = Not provided.
(DOCX)

S2 Table Non-human primate’s species infected by municipality and necropsy analysis. NA = not applicable; DENV = dengue virus, SLEV = Saint Louis encephalitis virus, ZIKV = Zika virus, YFV = Yellow fever virus.
(DOCX)

S3 Table Orthoflaviviruses found infecting NHPs according to the confirmed species by nucleotide sequencing of amplicons and sample type. DENV = dengue virus, SLEV = Saint Louis encephalitis virus.
(DOCX)

S4 Table Orthoflaviviruses positive NHPs according to each sampling county. (DOCX)

S5 Table Ecological and virological data of NHP. (XLSX)

S1 Fig Dengue Virus Typing tool from Genome Detective Version 4.1. Maximum likelihood tree of DENV3 based on our NS5 segment. Bootstrap values are indicated on the branches. The alignment was composed of 20 DENV-3 sequences with ~10.173 nucleotides, and the sequence of this study is stated as genotype III. The tree was inferred using the HKY85 + F nucleotide substitution model for all segments. Reference outgroup sequence for DENV3 (OK469356.1) was used. The detected fragment sequence in our study was deposited in GenBank: OQ032518.1. Trees were downloaded and re-plotted using phylogram representation to show ultra-fast bootstrap values.
(DOCX)

S2 Fig Dengue Virus Typing tool from Genome Detective Version 4.1. DENV3 Maximum likelihood tree of DENV3 based on our NS5 segment. The Alignment was composed of 19 sequences with ~10.173 nucleotides. All sequences belong to serotype 3, and the sequence of this study is stated as genotype III, major lineage C. The tree was inferred using the HKY85 + F nucleotide substitution model for all segments. Reference outgroup sequence for DENV3 (OK469356.1) was used. The detected fragment sequence in our study was deposited in GenBank: OQ032518.1. Trees were downloaded and re-plotted using phylogram representation to show ultra-fast bootstrap values.
(DOCX)

S3 Fig The serological results of the enzyme-linked immunosorbent assay (ELISA) for recombinant E2 (rE2) proteins of Mayaro virus (MAYV) and chikungunya virus (CHIKV) are presented. Panels (A) and (B) illustrate the assay outcomes for MAYV and CHIKV, respectively. The green chromogenic reaction observed in both assays indicates a positive IgG control reaction occurring in the upper half of the plate coated with rE2 proteins of MAYV (A) and CHIKV (B). In contrast, the lower half of the plate was coated with *Escherichia coli* (E. coli) as a negative control. The plates were incubated with 2,2’-azinobis(3-ethylbenzothiazoline-6-sulfonic acid) (ABTS) as the peroxidase substrate. Photograph taken by the author.
(DOCX)

S4 Fig Genomic fragments comparison among SLEV reference and sequenced samples. (A) Comparison of the alignment of these 11 samples revealed high homology between the fragments. Nucleotide variations were classified as silent mutations. Genomic fragments of sequences OQ290702 and OQ290699 exhibited a cytosine at position 8,427. Sequence OQ290699 displayed four additional nucleotide variations: a thymine instead of a cytosine at position 8,367, a guanine instead of adenine at position 8,379, and adenine instead of guanine at positions 8,382 and 8,412. Fragments form this study in blue shade. (B) Maximum likelihood tree of the *Orthoflavivirus* genus for SLEV based on the NS5 segment of 36 genomic fragments of NS5 protein. Bootstrap values are indicated on the branches. The tree was inferred by using the nucleotide substitution model GTR + F + G4 for all segments. Tree obtained with IQTree + Model finder, UFBootstrap and sh branch test. The viruses characterized in the study are shown in blue shade. The other sequences are from different regions of Brazil, Peru, Argentina, Trinidad and Tobago, Panamá and United States of America. The sequence in red corresponds to outgroup reference for SLEV. Sequences deposited in GenBank: OQ290696, OQ290697, OQ290698, OQ290699, OQ290700, OQ290701, OQ290702, OQ290703, OQ290704, OQ290705.
(DOCX)

S5 Fig Number of human cases of DENV in the cities of Ribeirão Preto and Catanduva. Dynamics of dengue virus (DENV) cases in Ribeirão Preto (blue square) and Catanduva (red triangle) from 2016 to 2022. Human cases increased in 2019, peaked in 2020, and receded in 2021, in both cities. This trend correlates with non-human primate (NHP) cases observed during the same period.
(DOCX)

S6 Fig Number of human cases of ZIKV in the cities of Ribeirão Preto and Catanduva. Dynamics of Zika virus (ZIKV) cases in Ribeirão Preto (blue square) and Catanduva (red triangle) from 2016 to 2021. Human cases increased in 2017, and decreased in 2018, peaked in 2019, and receded in 2020 and 2021, in Ribeirão Preto city. There were no human ZIKV cases reported in Catanduva.
(DOCX)

S7 Fig Number of human cases of CHIKV in the cities of Ribeirão Preto and Catanduva. Dynamics of Chikungunya virus (CHIKV) cases in Ribeirão Preto (blue square) and Catanduva (red triangle) from 2016 to 2021. Human cases increased in 2017 in both cities, and decreased in 2018–2020, with a small increase in 2021 in Ribeirão Preto city, but not Catanduva city. There were no human CHIKV cases reported in Catanduva city in 2018 and 2019.
(DOCX)
