# Supplementary material for: Eco-epidemiology of arbovirus infections among non-human primates in Southeastern Brazil
Source: PLoS Negl Trop Dis. 2025 Nov 19;19(11):e0013743. doi: 10.1371/journal.pntd.0013743 (PMC12643272; doi:10.1371/journal.pntd.0013743)
Supplement: S1 Table — M-N = Multi nested, N**(-) = Nested primer, (+) = Forward primer, (-) = Reverse primer, NS = Non structural protein, r = Recombinant antigen, NP = Not provided. (DOCX) [file pntd.0013743.s001.docx]

**Eco-epidemiology of arbovirus infections among non-human primates in southeastern Brazil**

**Short title: Arbovirus eco-epidemiology in non-human primates**

Leonardo La Serra^1^*, Rafael L. S. Cazarotti^1^, Vitoria M. Scrich^2^, Larissa M. Bueno^3^, Andreia N. Carvalho^4^, Daniel M. M. Jorge^5,1^, Murilo H. A. Cassiano^4,1^, Renan B. do Amaral^1^, Soraya J. Badra^1^, Gustavo R. Canale^6^, Gilberto Sabino-Santos^1,7,8^ *^¶^ and Luiz T. M. Figueiredo^1¶^

^1^ Center for Virology Research, Ribeirão Preto Medical School, University of São Paulo, Ribeirão Preto, São Paulo, Brazil.

^2^ Environmental Sciences Graduate Program, Institute of Energy and Environment, University of Sao Paulo, Ubatuba, Brazil.

^3^ Department of Veterinary Medicine, University of São Paulo, Pirassununga, São Paulo, Brazil

^4^ Department of Cellular and Molecular Biology and Pathogenic Bioagent, University of São Paulo, Ribeirão Preto, São Paulo, Brazil

^5^ Department of Microbiology and Immunology, University of Michigan Medical School, Ann Arbor, Michigan, United States of America

^6^ Institute of Natural, Human, and Social Sciences, Federal University of Mato Grosso, Sinop, Mato Grosso, Brazil

^7^ Department of Microbiology & Immunology, Tulane University School of Medicine, New Orleans, Louisiana, United States of America

^8^ Smithsonian Institution, National Zoo and Conservation Biology Institute, Front Royal, Virginia, United States of America

*laserra@usp.br (LLS), [sabinosantosg@si.edu](mailto:gsabino@scripps.edu)/gsabino@tulane.edu (GSS)

^¶^These senior authors contributed equally to this article.

**S1 Table.** Sequence of primers for RT-PCR, RT-qPCR and the ELISA antigens used in our study.

| **Assay** | **Primer name / Virus** | **Primer/Probe sequence (5' -3') / Target** | **Gene** | **Reference** |
| --- | --- | --- | --- | --- |
| M-N-PCR orthoflavivirus | FG1 (+) | TCAAGGAACTCCACACATGAGATGTACT | NS5 | [36] |
|  | NYF (-) | TCAGAAGACCAAGAGGTCATGT |  |  |
|  | NDEN1 (-) | CGTTTTGCTCTTGTGTGCGC |  |  |
|  | NDEN2 (-) | GAACCAGTTTGTTTDRTTTCATAGCTGCC |  |  |
|  | NDEN3 (-) | CCCATTGGTTCTCCTCTGTG |  |  |
|  | NDEN4 (-) | GCAATCGCTGAAGCCTTCTCCC |  |  |
|  | CZIKA120 (-) | CACTGGCCTCCTAGGCCCGTCCAT |  |  |
|  | NSLE (-) | ATTCTTCTCTCAATCTCCGT |  |  |
|  | NILH (-) | TCCACCGCTGATCTGAGCCCGTGA |  |  |
|  | NROC (-) | TCACTCTTCAGCCTTTCG |  |  |
|  | NWN (-) | TGGTGTCTGAGTTGAGCAGGG |  |  |
|  |  |  |  |  |
| RT - qPCR Chikungunya virus | CHIKV (+) | GACAATGCGCGCGGTACC | NS1 | [38] |
|  | CHIKV (-) | TGTTGTTTTGTGGCGCCT |  |  |
|  | CHIKV probe | FAM-GCCACGCAGGAATCGGAAGAATAAGAAGC-TAMRA |  |  |
|  |  |  |  |  |
| RT - qPCR Mayaro virus | MAYV (+) | AAGCTCTTCCTCTGCATTGC | NS1 | [37] |
|  | MAYV 1 (-) | TGCTGGAAACGCTCTCTGTA |  |  |
|  | MAYV 2 (-) | TGCTGGAAATGCTCTTTGTA |  |  |
|  | MAYV probe | FAM-GCCGAGAGCCCGTTTTTAAAATCAC-BHQ1 |  |  |
|  |  |  |  |  |
| ELISA | CHIKV | rE2 of CHIKV |  | [39] |
|  | MAYV | rE2 of MAYV |  | [40] |
| M-N= Multi nested, N**(-) = Nested primer, (+) = Forward primer, (-) = Reverse primer, NS = Non structural protein, r= Recombinant antigen, | | | | |
| NP = Not provided |  |  |  |  |
