# Supplementary material for: Eco-epidemiology of arbovirus infections among non-human primates in Southeastern Brazil
Source: PLoS Negl Trop Dis. 2025 Nov 19;19(11):e0013743. doi: 10.1371/journal.pntd.0013743 (PMC12643272; doi:10.1371/journal.pntd.0013743)
Supplement: S2 Table — NA = not applicable; DENV = dengue virus, SLEV = Saint Louis encephalitis virus, ZIKV = Zika virus, YFV = Yellow fever virus. (DOCX) [file pntd.0013743.s002.docx]

**Eco-epidemiology of arbovirus infections among non-human primates in southeastern Brazil**

**Short title: Arbovirus eco-epidemiology in non-human primates**

Leonardo La Serra^1^*, Rafael L. S. Cazarotti^1^, Vitoria M. Scrich^2^, Larissa M. Bueno^3^, Andreia N. Carvalho^4^, Daniel M. M. Jorge^5,1^, Murilo H. A. Cassiano^4,1^, Renan B. do Amaral^1^, Soraya J. Badra^1^, Gustavo R. Canale^6^, Gilberto Sabino-Santos^1,7,8^ *^¶^ and Luiz T. M. Figueiredo^1¶^

^1^ Center for Virology Research, Ribeirão Preto Medical School, University of São Paulo, Ribeirão Preto, São Paulo, Brazil.

^2^ Environmental Sciences Graduate Program, Institute of Energy and Environment, University of Sao Paulo, Ubatuba, Brazil.

^3^ Department of Veterinary Medicine, University of São Paulo, Pirassununga, São Paulo, Brazil

^4^ Department of Cellular and Molecular Biology and Pathogenic Bioagent, University of São Paulo, Ribeirão Preto, São Paulo, Brazil

^5^ Department of Microbiology and Immunology, University of Michigan Medical School, Ann Arbor, Michigan, United States of America

^6^ Institute of Natural, Human, and Social Sciences, Federal University of Mato Grosso, Sinop, Mato Grosso, Brazil

^7^ Department of Microbiology & Immunology, Tulane University School of Medicine, New Orleans, Louisiana, United States of America

^8^ Smithsonian Institution, National Zoo and Conservation Biology Institute, Front Royal, Virginia, United States of America

*[laserra@usp.br](mailto:laserra@usp.br) (LLS), [sabinosantosg@si.edu](mailto:gsabino@scripps.edu)/gsabino@tulane.edu (GSS)

^¶^These senior authors contributed equally to this article.

| **S2 Table.** Non-human primate’s species infected by municipality and necropsy analysis.   \| Species \| County \| Necropsy (Clinical Evaluation) \| Virus \| \| --- \| --- \| --- \| --- \| \| *Callithrix penicillata* \| Ribeirão Preto \| Cachexia, adult male. abrasions, fractures and severe bleeding. Possible hit by a vehicle. \| SLEV \| \| *Callithrix penicillata* \| Ribeirão Preto \| Normal body condition. No signs of abrasions. Congested internal organs and a large volume of blood. Possible poisoning. \| ZIKV \| \| *Alouatta caraya* \| Ribeirão Preto \| Normal physical state. Evidence of abrasions and injuries. Possible electrocution. \| SLEV/ZIKV \| \| *Callithrix penicillata* \| Ribeirão Preto \| Normal body condition. No signs of abrasions. Intense bleeding in the abdominal cavity. \| DENV3 \| \| *Callithrix penicillata* \| Ribeirão Preto \| Normal body condition. Signs of abrasions and injuries. Probable dog attack. \| SLEV \| \| *Callithrix penicillata* \| Ribeirão Preto \| Normal body condition. Signs of abrasions and injuries. Normal internal organs. Death to be clarified \| ZIKV \| \| *Callithrix penicillata* \| Ribeirão Preto \| Normal body condition. Friable liver, splenomegaly, brownish color, and necrotic spots. Decreased kidneys and lungs. Hypocreptant lungs with necrotic borders. Organs adhered to the wall and a large amount of fibrous tissue. \| DENV1 \| \| *Callithrix penicillata* \| Ribeirão Preto \| Normal body condition. Signs of abrasions and injuries. Normal internal organs. Death due to chest trauma. \| ZIKV \| \| *Callithrix penicillata* \| Ribeirão Preto \| Normal body condition. Signs of abrasions and injuries. Hematoma in the skull. Internal organs normal. Death due to cranial trauma. \| ZIKV/DENV3 \| \| *Callithrix penicillata* \| Ribeirão Preto \| Normal body condition. Hematoma in the skull. Death due to organ rupture and skull crushing by a traffic accident. \| ZIKV \| \| *Callithrix penicillata* \| Ribeirão Preto \| Normal body condition. Congested liver and stomach. Liver with necrotic edges and empty gallbladder. Hemorrhagic and decreased lungs volume. \| ZIKV \| \| *Callithrix penicillata* \| São Carlos \| NA \| SLEV \| \| *Callithrix penicillata* \| São Carlos \| NA \| YFV \| \| *Saguinus midas* \| São Carlos \| NA \| SLEV \| \| *Saguinus midas* \| São Carlos \| NA \| SLEV \| \| *Alouatta caraya* \| Catanduva \| NA \| SLEV \| \| *Alouatta caraya* \| Catanduva \| NA \| SLEV/ZIKV \| \| *Callithrix penicillata* \| Catanduva \| NA \| SLEV \| \| *Callithrix penicillata* \| Catanduva \| NA \| SLEV \| \| *Callithrix penicillata* \| Catanduva \| NA \| SLEV \| \| *Callithrix penicillata* \| Catanduva \| NA \| SLEV \| \| *Sapajus apella* \| Catanduva \| NA \| DENV1 \| \| *Sapajus apella* \| Catanduva \| NA \| SLEV \| \| *Sapajus apella* \| Catanduva \| NA \| SLEV \| \| *Sapajus apella* \| Catanduva \| NA \| SLEV \| \| *Sapajus apella* \| Catanduva \| NA \| DENV2 \| \| *Sapajus apella* \| Catanduva \| NA \| SLEV \| \| *Callithrix penicillata* \| Ribeirão Preto \| Cachexia, with lesions on the tail and head. Death due to trauma from aggression. \| ZIKV \| \| Callithrix penicillata \| Ribeirão Preto \| Apparently healthy animal. Severe burns. Death due to electric shock. \| ZIKV \| \| Alouatta caraya \| Ribeirão Preto \| Normal body condition. Internal organs had normal appearance. Cranial trauma and burns present. Death caused by trauma and burns from high-voltage wire and fall. \| ZIKV \| \| NA = not applicable; DENV = dengue virus , SLEV = Saint Louis encephalitis virus, ZIKV = Zika virus, YFV = Yellow fever virus \| \| \| \| |
| --- | --- | --- | --- | --- | --- | --- | --- | --- | --- | --- | --- | --- | --- | --- | --- | --- | --- | --- | --- | --- | --- | --- | --- | --- | --- | --- | --- | --- | --- | --- | --- | --- | --- | --- | --- | --- | --- | --- | --- | --- | --- | --- | --- | --- | --- | --- | --- | --- | --- | --- | --- | --- | --- | --- | --- | --- | --- | --- | --- | --- | --- | --- | --- | --- | --- | --- | --- | --- | --- | --- | --- | --- | --- | --- | --- | --- | --- | --- | --- | --- | --- | --- | --- | --- | --- | --- | --- | --- | --- | --- | --- | --- | --- | --- | --- | --- | --- | --- | --- | --- | --- | --- | --- | --- | --- | --- | --- | --- | --- | --- | --- | --- | --- | --- | --- | --- | --- | --- | --- | --- | --- | --- | --- | --- | --- | --- | --- | --- |
