# Supplementary material for: Eco-epidemiology of arbovirus infections among non-human primates in Southeastern Brazil
Source: PLoS Negl Trop Dis. 2025 Nov 19;19(11):e0013743. doi: 10.1371/journal.pntd.0013743 (PMC12643272; doi:10.1371/journal.pntd.0013743)
Supplement: S3 Table — DENV = dengue virus, SLEV = Saint Louis encephalitis virus. (DOCX) [file pntd.0013743.s003.docx]

**Eco-epidemiology of arbovirus infections among non-human primates in southeastern Brazil**

**Short title: Arbovirus eco-epidemiology in non-human primates**

Leonardo La Serra^1^*, Rafael L. S. Cazarotti^1^, Vitoria M. Scrich^2^, Larissa M. Bueno^3^, Andreia N. Carvalho^4^, Daniel M. M. Jorge^5,1^, Murilo H. A. Cassiano^4,1^, Renan B. do Amaral^1^, Soraya J. Badra^1^, Gustavo R. Canale^6^, Gilberto Sabino-Santos^1,7,8^ *^¶^ and Luiz T. M. Figueiredo^1¶^

^1^ Center for Virology Research, Ribeirão Preto Medical School, University of São Paulo, Ribeirão Preto, São Paulo, Brazil.

^2^ Environmental Sciences Graduate Program, Institute of Energy and Environment, University of Sao Paulo, Ubatuba, Brazil.

^3^ Department of Veterinary Medicine, University of São Paulo, Pirassununga, São Paulo, Brazil

^4^ Department of Cellular and Molecular Biology and Pathogenic Bioagent, University of São Paulo, Ribeirão Preto, São Paulo, Brazil

^5^ Department of Microbiology and Immunology, University of Michigan Medical School, Ann Arbor, Michigan, United States of America

^6^ Institute of Natural, Human, and Social Sciences, Federal University of Mato Grosso, Sinop, Mato Grosso, Brazil

^7^ Department of Microbiology & Immunology, Tulane University School of Medicine, New Orleans, Louisiana, United States of America

^8^ Smithsonian Institution, National Zoo and Conservation Biology Institute, Front Royal, Virginia, United States of America

*laserra@usp.br (LLS), [sabinosantosg@si.edu](mailto:gsabino@scripps.edu)/gsabino@tulane.edu (GSS)

^¶^These senior authors contributed equally to this article.

**S3 Table.** Orthoflaviviruses found infecting NHPs according to the confirmed species by nucleotide sequencing of amplicons and sample type.

| **Virus** | **N** | | **Species** | | **Positive Samples** | **Fragment length** |  |
| --- | --- | --- | --- | --- | --- | --- | --- |
| DENV 3 | 1 | *Callithrix penicillata* | | Blood | | 387 | |
| SLEV | 1 | *Alouatta caraya* | | Blood, saliva | | 102 | |
|  | 4 | *Callithrix penicillata* | | Blood, saliva and feces | | 26, 55, 151, 173 | |
|  | 1 | *Saguinus midas* | | Saliva | | 133 | |
|  | 4 | *Sapajus apella* | | Blood, saliva, urine | | 93, 98, 101, 113 | |

DENV= dengue virus, SLEV= Saint Louis encephalitis virus
