# Supplementary material for: Eco-epidemiology of arbovirus infections among non-human primates in Southeastern Brazil
Source: PLoS Negl Trop Dis. 2025 Nov 19;19(11):e0013743. doi: 10.1371/journal.pntd.0013743 (PMC12643272; doi:10.1371/journal.pntd.0013743)
Supplement: S1 Fig — Maximum likelihood tree of DENV3 based on our NS5 segment. Bootstrap values are indicated on the branches. The alignment was composed of 20 DENV-3 sequences with ~10.173 nucleotides, and the sequence of this study is stated as genotype III. The tree was inferred using the HKY85 + F nucleotide substitution model for all segments. Reference outgroup sequence for DENV3 (OK469356.1) was used. The detected fragment sequence in our study was deposited in GenBank: OQ032518.1. Trees were downloaded and re-plotted using phylogram representation to show ultra-fast bootstrap values. (DOCX) [file pntd.0013743.s006.docx]

**Eco-epidemiology of arbovirus infections among non-human primates in southeastern Brazil**

**Short title: Arbovirus eco-epidemiology in non-human primates**

Leonardo La Serra^1^*, Rafael L. S. Cazarotti^1^, Vitoria M. Scrich^2^, Larissa M. Bueno^3^, Andreia N. Carvalho^4^, Daniel M. M. Jorge^5,1^, Murilo H. A. Cassiano^4,1^, Renan B. do Amaral^1^, Soraya J. Badra^1^, Gustavo R. Canale^6^, Gilberto Sabino-Santos^1,7,8^ *^¶^ and Luiz T. M. Figueiredo^1¶^

^1^ Center for Virology Research, Ribeirão Preto Medical School, University of São Paulo, Ribeirão Preto, São Paulo, Brazil.

^2^ Environmental Sciences Graduate Program, Institute of Energy and Environment, University of Sao Paulo, Ubatuba, Brazil.

^3^ Department of Veterinary Medicine, University of São Paulo, Pirassununga, São Paulo, Brazil

^4^ Department of Cellular and Molecular Biology and Pathogenic Bioagent, University of São Paulo, Ribeirão Preto, São Paulo, Brazil

^5^ Department of Microbiology and Immunology, University of Michigan Medical School, Ann Arbor, Michigan, United States of America

^6^ Institute of Natural, Human, and Social Sciences, Federal University of Mato Grosso, Sinop, Mato Grosso, Brazil

^7^ Department of Microbiology & Immunology, Tulane University School of Medicine, New Orleans, Louisiana, United States of America

^8^ Smithsonian Institution, National Zoo and Conservation Biology Institute, Front Royal, Virginia, United States of America

*laserra@usp.br (LLS), [sabinosantosg@si.edu](mailto:gsabino@scripps.edu)/gsabino@tulane.edu (GSS)

^¶^These senior authors contributed equally to this article.

**S1 Fig. Dengue Virus Typing tool from Genome Detective Version 4.1.** Maximum-likelihood tree of DENV-3 based on our NS5 segment. Bootstrap values are indicated on the branches. The alignment was composed of 20 DENV-3 sequences with ~10.173 nucleotides, and the sequence of this study is stated as genotype III. The tree was inferred using the HKY85+F nucleotide substitution model for all segments. The reference outgroup sequence for DENV3 (OK469356.1) was used. The fragment sequence detected in our study was deposited in GenBank under accession OQ032518.1. Trees were downloaded and re-plotted using phylogram representation to show ultra-fast bootstrap values.
