# Supplementary material for: Eco-epidemiology of arbovirus infections among non-human primates in Southeastern Brazil
Source: PLoS Negl Trop Dis. 2025 Nov 19;19(11):e0013743. doi: 10.1371/journal.pntd.0013743 (PMC12643272; doi:10.1371/journal.pntd.0013743)
Supplement: S3 Fig — Panels (A) and (B) illustrate the assay outcomes for MAYV and CHIKV, respectively. The green chromogenic reaction observed in both assays indicates a positive IgG control reaction occurring in the upper half of the plate coated with rE2 proteins of MAYV (A) and CHIKV (B). In contrast, the lower half of the plate was coated with Escherichia coli (E. coli) as a negative control. The plates were incubated with 2,2’-azinobis(3-ethylbenzothiazoline-6-sulfonic acid) (ABTS) as the peroxidase substrate. Photograph taken by the author. (DOCX) [file pntd.0013743.s008.docx]

**Eco-epidemiology of arbovirus infections among non-human primates in southeastern Brazil**

**Short title: Arbovirus eco-epidemiology in non-human primates**

Leonardo La Serra^1^*, Rafael L. S. Cazarotti^1^, Vitoria M. Scrich^2^, Larissa M. Bueno^3^, Andreia N. Carvalho^4^, Daniel M. M. Jorge^5,1^, Murilo H. A. Cassiano^4,1^, Renan B. do Amaral^1^, Soraya J. Badra^1^, Gustavo R. Canale^6^, Gilberto Sabino-Santos^1,7,8^ *^¶^ and Luiz T. M. Figueiredo^1¶^

^1^ Center for Virology Research, Ribeirão Preto Medical School, University of São Paulo, Ribeirão Preto, São Paulo, Brazil.

^2^ Environmental Sciences Graduate Program, Institute of Energy and Environment, University of Sao Paulo, Ubatuba, Brazil.

^3^ Department of Veterinary Medicine, University of São Paulo, Pirassununga, São Paulo, Brazil

^4^ Department of Cellular and Molecular Biology and Pathogenic Bioagent, University of São Paulo, Ribeirão Preto, São Paulo, Brazil

^5^ Department of Microbiology and Immunology, University of Michigan Medical School, Ann Arbor, Michigan, United States of America

^6^ Institute of Natural, Human, and Social Sciences, Federal University of Mato Grosso, Sinop, Mato Grosso, Brazil

^7^ Department of Microbiology & Immunology, Tulane University School of Medicine, New Orleans, Louisiana, United States of America

^8^ Smithsonian Institution, National Zoo and Conservation Biology Institute, Front Royal, Virginia, United States of America

*laserra@usp.br (LLS), [sabinosantosg@si.edu](mailto:gsabino@scripps.edu)/gsabino@tulane.edu (GSS)

^¶^These senior authors contributed equally to this article.

**S3 Fig.** **The serological results of the enzyme-linked immunosorbent assay (ELISA) for recombinant E2 (rE2) proteins of Mayaro virus (MAYV) and chikungunya virus (CHIKV) are presented.** Panels (A) and (B) illustrate the assay outcomes for MAYV and CHIKV, respectively. The green chromogenic reaction observed in both assays indicates a positive IgG control in the upper half of the plate coated with rE2 proteins of MAYV (A) and CHIKV (B). In contrast, the lower half of the plate was coated with *Escherichia coli* (E. coli) as a negative control. The plates were incubated with 2,2’-azinobis(3-ethylbenzothiazoline-6-sulfonic acid) (ABTS) as the peroxidase substrate. Photograph taken by the authors.
