# Supplementary material for: Eco-epidemiology of arbovirus infections among non-human primates in Southeastern Brazil
Source: PLoS Negl Trop Dis. 2025 Nov 19;19(11):e0013743. doi: 10.1371/journal.pntd.0013743 (PMC12643272; doi:10.1371/journal.pntd.0013743)
Supplement: S4 Fig — (A) Comparison of the alignment of these 11 samples revealed high homology between the fragments. Nucleotide variations were classified as silent mutations. Genomic fragments of sequences OQ290702 and OQ290699 exhibited a cytosine at position 8,427. Sequence OQ290699 displayed four additional nucleotide variations: a thymine instead of a cytosine at position 8,367, a guanine instead of adenine at position 8,379, and adenine instead of guanine at positions 8,382 and 8,412. Fragments form this study in blue shade. (B) Maximum likelihood tree of the Orthoflavivirus genus for SLEV based on the NS5 segment of 36 genomic fragments of NS5 protein. Bootstrap values are indicated on the branches. The tree was inferred by using the nucleotide substitution model GTR + F + G4 for all segments. Tree obtained with IQTree + Model finder, UFBootstrap and sh branch test. The viruses characterized in the study are shown in blue shade. The other sequences are from different regions of Brazil, Peru, Argentina, Trinidad and Tobago, Panamá and United States of America. The sequence in red corresponds to outgroup reference for SLEV. Sequences deposited in GenBank: OQ290696, OQ290697, OQ290698, OQ290699, OQ290700, OQ290701, OQ290702, OQ290703, OQ290704, OQ290705. (DOCX) [file pntd.0013743.s009.docx]

**Eco-epidemiology of arbovirus infections among non-human primates in southeastern Brazil**

**Short title: Arbovirus eco-epidemiology in non-human primates**

Leonardo La Serra^1^*, Rafael L. S. Cazarotti^1^, Vitoria M. Scrich^2^, Larissa M. Bueno^3^, Andreia N. Carvalho^4^, Daniel M. M. Jorge^5,1^, Murilo H. A. Cassiano^4,1^, Renan B. do Amaral^1^, Soraya J. Badra^1^, Gustavo R. Canale^6^, Gilberto Sabino-Santos^1,7,8^ *^¶^ and Luiz T. M. Figueiredo^1¶^

^1^ Center for Virology Research, Ribeirão Preto Medical School, University of São Paulo, Ribeirão Preto, São Paulo, Brazil.

^2^ Environmental Sciences Graduate Program, Institute of Energy and Environment, University of Sao Paulo, Ubatuba, Brazil.

^3^ Department of Veterinary Medicine, University of São Paulo, Pirassununga, São Paulo, Brazil

^4^ Department of Cellular and Molecular Biology and Pathogenic Bioagent, University of São Paulo, Ribeirão Preto, São Paulo, Brazil

^5^ Department of Microbiology and Immunology, University of Michigan Medical School, Ann Arbor, Michigan, United States of America

^6^ Institute of Natural, Human, and Social Sciences, Federal University of Mato Grosso, Sinop, Mato Grosso, Brazil

^7^ Department of Microbiology & Immunology, Tulane University School of Medicine, New Orleans, Louisiana, United States of America

^8^ Smithsonian Institution, National Zoo and Conservation Biology Institute, Front Royal, Virginia, United States of America

*laserra@usp.br (LLS), [sabinosantosg@si.edu](mailto:gsabino@scripps.edu)/gsabino@tulane.edu (GSS)

^¶^These senior authors contributed equally to this article.

**
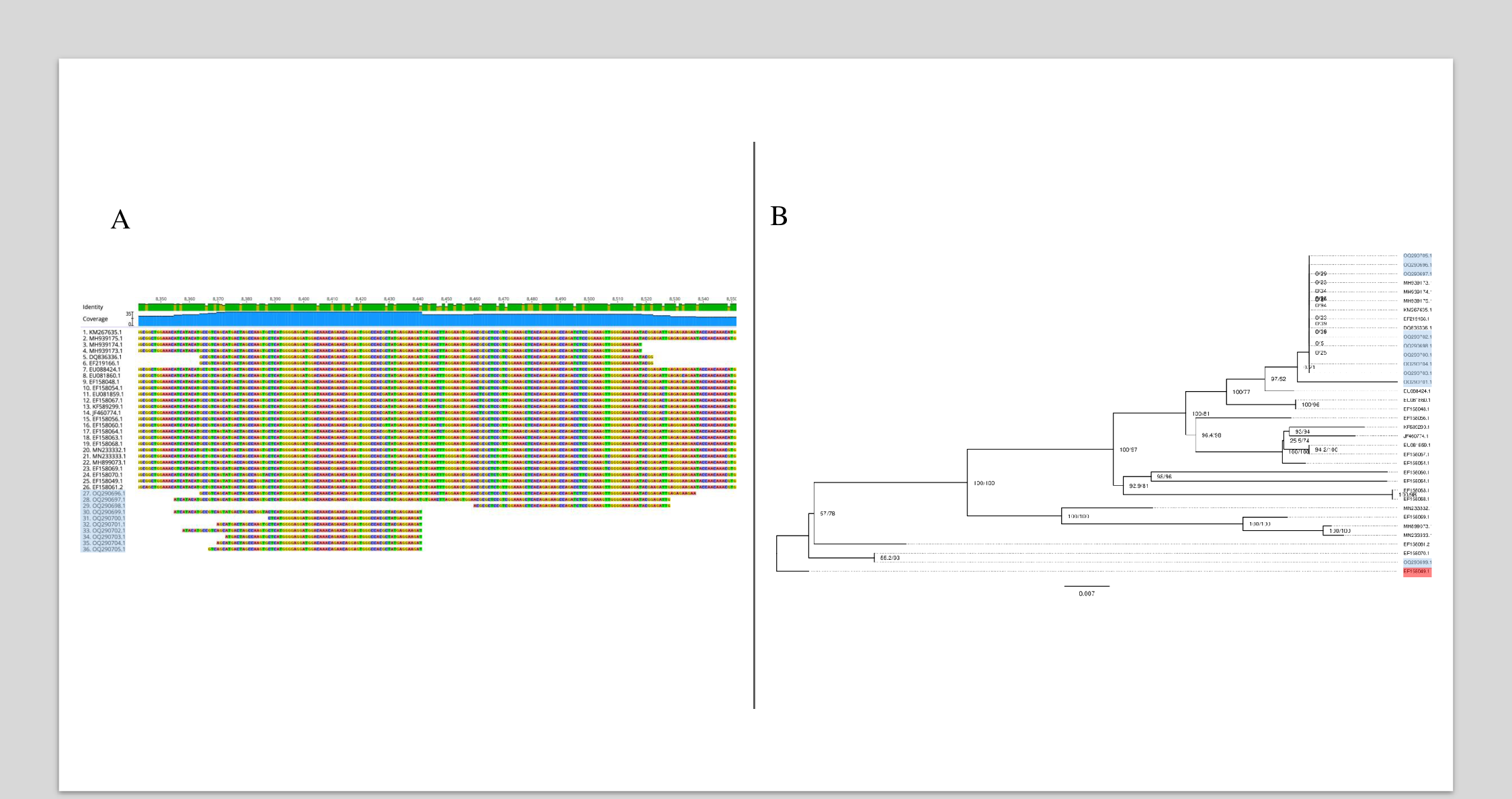
**

**S4 Fig. Genomic fragments comparison of *Orthoflavivirus louisense* (SLEV) reference with sequenced SLEV samples in our study.** (A) Comparison of the alignment of these 11 samples revealed high homology between the fragments. Nucleotide variations were classified as silent mutations. Genomic fragments of sequences OQ290702 and OQ290699 exhibited a cytosine at position 8,427. Sequence OQ290699 displayed four additional nucleotide variations: a thymine instead of a cytosine at position 8,367, a guanine instead of adenine at position 8,379, and adenine instead of guanine at positions 8,382 and 8,412. Fragments form this study in blue shade. (B) Maximum likelihood tree of the *Orthoflavivirus* genus for SLEV based on the NS5 segment of 36 genomic fragments of NS5 protein. Bootstrap values are indicated on the branches. The tree was inferred by using the nucleotide substitution model GTR+F+G4 for all segments. Tree obtained with IQTree + Model finder, UFBootstrap and sh branch test. The viruses characterized in the study are shown in blue shade. The other sequences are from different regions of Brazil, Peru, Argentina, Trinidad and Tobago, Panamá and United States of America. The sequence in red corresponds to outgroup reference for SLEV. Sequences deposited in GenBank: OQ290696, OQ290697, OQ290698, OQ290699, OQ290700, OQ290701, OQ290702, OQ290703, OQ290704, OQ290705.
