# Supplementary material for: Eco-epidemiology of arbovirus infections among non-human primates in Southeastern Brazil
Source: PLoS Negl Trop Dis. 2025 Nov 19;19(11):e0013743. doi: 10.1371/journal.pntd.0013743 (PMC12643272; doi:10.1371/journal.pntd.0013743)
Supplement: S6 Fig — Dynamics of Zika virus (ZIKV) cases in Ribeirão Preto (blue square) and Catanduva (red triangle) from 2016 to 2021. Human cases increased in 2017, and decreased in 2018, peaked in 2019, and receded in 2020 and 2021, in Ribeirão Preto city. There were no human ZIKV cases reported in Catanduva. (DOCX) [file pntd.0013743.s011.docx]

**Eco-epidemiology of arbovirus infections among non-human primates in southeastern Brazil**

**Short title: Arbovirus eco-epidemiology in non-human primates**

Leonardo La Serra^1^*, Rafael L. S. Cazarotti^1^, Vitoria M. Scrich^2^, Larissa M. Bueno^3^, Andreia N. Carvalho^4^, Daniel M. M. Jorge^5,1^, Murilo H. A. Cassiano^4,1^, Renan B. do Amaral^1^, Soraya J. Badra^1^, Gustavo R. Canale^6^, Gilberto Sabino-Santos^1,7,8^ *^¶^ and Luiz T. M. Figueiredo^1¶^

^1^ Center for Virology Research, Ribeirão Preto Medical School, University of São Paulo, Ribeirão Preto, São Paulo, Brazil.

^2^ Environmental Sciences Graduate Program, Institute of Energy and Environment, University of Sao Paulo, Ubatuba, Brazil.

^3^ Department of Veterinary Medicine, University of São Paulo, Pirassununga, São Paulo, Brazil

^4^ Department of Cellular and Molecular Biology and Pathogenic Bioagent, University of São Paulo, Ribeirão Preto, São Paulo, Brazil

^5^ Department of Microbiology and Immunology, University of Michigan Medical School, Ann Arbor, Michigan, United States of America

^6^ Institute of Natural, Human, and Social Sciences, Federal University of Mato Grosso, Sinop, Mato Grosso, Brazil

^7^ Department of Microbiology & Immunology, Tulane University School of Medicine, New Orleans, Louisiana, United States of America

^8^ Smithsonian Institution, National Zoo and Conservation Biology Institute, Front Royal, Virginia, United States of America

*laserra@usp.br (LLS), [sabinosantosg@si.edu](mailto:gsabino@scripps.edu)/gsabino@tulane.edu (GSS)

^¶^These senior authors contributed equally to this article.

**S6 Fig. Number of human cases of ZIKV in the cities of Ribeirão Preto and Catanduva.** Dynamics of Zika virus (ZIKV) cases in Ribeirão Preto (blue square) and Catanduva (red triangle) from 2016 to 2021. Human cases increased in 2017, and decreased in 2018, peaked in 2019, and receded in 2020 and 2021, in Ribeirão Preto city. There were no human ZIKV cases reported in Catanduva.
